# Supplementary material for: Cardioprotective Effects of Dexmedetomidine in an Oxidative-Stress In Vitro Model of Neonatal Rat Cardiomyocytes
Source: Antioxidants (Basel). 2023 Jun 2;12(6):1206. doi: 10.3390/antiox12061206 (PMC10295527; doi:10.3390/antiox12061206)
Supplement: Supplementary file 1 [file antioxidants-12-01206-s001.zip › Table S1 Quantification of proliferation1.pdf]

Table S1. Quantification of co-labeled Ki67-/ α-actinin cardiomyocytes (H9c2 and NRCM)

**Hypoxia (5% O_2)**					
**dexmedetomidine**		**–**	**0.1 µM**	**1 µM**	**10 µM**
**Ki67+**	**H9c2**	49±4.3	80±5.2	83±4.3	66±5.1
**Hypertrophy**	**H9c2**	111±4.7	94±5.2	92±6.2	100±4.7
**CycD2**	**H9c2**	57±4.4	78±6.2	91±5.8	96±4.8
**CycD2**	**NRCM**	61±2.4	77±8.8	96±8.0	95±6.6
**Normoxia (21% O_2)**					
**dexmedetomidine**		**–**	**0.1 µM**	**1 µM**	**10 µM**
**Ki67+**	**H9c2**	100±10.9	117±8.1	112±11.4	99±10.4
**Hypertrophy**	**H9c2**	100±3.7	87±3.2	94±5.8	115±5.7
**CycD2**	**H9c2**	100±0.0	79±7.9	81±5.8	99±3.1
**CycD2**	**NRCM**	100±0.0	81±4.5	90±7.6	80±5.2
**Hyperoxia (80% O_2)**					
**dexmedetomidine**		**–**	**0.1 µM**	**1 µM**	**10 µM**
**Ki67+**	**H9c2**	42±8.8	60±6.1	59±7.1	48±6.1
**Hypertrophy**	**H9c2**	209±21.9	196±24.9	184±28.3	203±20.6
**CycD2**	**H9c2**	66±4.7	96±6.4	110±5.9	109±4.2
**CycD2**	**NRCM**	57±5.5	84±13.8	84±4.1	95±2.4

Data are normalized to the level of cardiomyocytes exposed to normoxia (100%) and are presented as mean (%) ± standard error of the mean (SEM). n = 6 individual experiments/group.
