# Supplementary material for: Cardioprotective Effects of Dexmedetomidine in an Oxidative-Stress In Vitro Model of Neonatal Rat Cardiomyocytes
Source: Antioxidants (Basel). 2023 Jun 2;12(6):1206. doi: 10.3390/antiox12061206 (PMC10295527; doi:10.3390/antiox12061206)
Supplement: Supplementary file 1 [file antioxidants-12-01206-s001.zip › Table S2 Quantitation of cytotoxicity.pdf]

Table S2. Quantification of cytotoxicity (H9c2 and NRCM)

**Hypoxia (5% O_2)**					
**dexmedetomidine**		**–**	**0.1 µM**	**1 µM**	**10 µM**
**LDH**	**H9c2**	120±10.9	109±7.4	105±5.7	118±1.9
**LDH**	**NRCM**	102±6.1	89±2.9	97±7.5	83±8.1
**CCK-8**	**H9c2**	90±3.9	89±2.4	83±3.5	84±3.2
**Normoxia (21% O_2)**					
**dexmedetomidine**		**–**	**0.1 µM**	**1 µM**	**10 µM**
**LDH**	**H9c2**	100±0.0	84±0.7	91±2.8	92±2.8
**LDH**	**NRCM**	100±0.0	91±4.9	87±7.5	88±3.0
**CCK-8**	**H9c2**	100±1.9	92±2.4	90±1.4	93±1.8
**Hyperoxia (80% O_2)**					
**dexmedetomidine**		**–**	**0.1 µM**	**1 µM**	**10 µM**
**LDH**	**H9c2**	120±6.7	106±4.4	103±3.7	106±3.9
**LDH**	**NRCM**	192±5.4	157±21.4	174±8.5	164±20.8
**CCK-8**	**H9c2**	77±3.1	81±2.5	71±2.9	73±3.0

Data are normalized to the level of cardiomyocytes exposed to normoxia (100%) and are presented as mean (%) ± standard error of the mean (SEM). n = 6 individual experiments/group.
