# Supplementary material for: Cardioprotective Effects of Dexmedetomidine in an Oxidative-Stress In Vitro Model of Neonatal Rat Cardiomyocytes
Source: Antioxidants (Basel). 2023 Jun 2;12(6):1206. doi: 10.3390/antiox12061206 (PMC10295527; doi:10.3390/antiox12061206)
Supplement: Supplementary file 1 [file antioxidants-12-01206-s001.zip › Table S3 Quantification of apoptosis.pdf]

Table S3. Quantification of apoptosis factors (H9c2 and NRCM)

**Hypoxia (5% O_2)**					
**dexmedetomidine**		**–**	**0.1 µM**	**1 µM**	**10 µM**
**Casp3**	**H9c2**	153±5.6	86±3.5	89±5.4	82±7.6
**Casp3**	**NRCM**	127±3.0	66±3.4	74±7.2	81±3.7
**Casp8**	**H9c2**	134±10.8	69±9.8	76±12.4	74±11.4
**Casp8**	**NRCM**	150±15.7	80±6.3	83±6.5	85±10.0
**AIF**	**H9c2**	142±8.1	78±8.6	104±7.5	78±8.1
**AIF**	**NRCM**	45±5.5	54±3.7	73±8.0	71±3.7
**Normoxia (21% O_2)**					
**dexmedetomidine**		**–**	**0.1 µM**	**1 µM**	**10 µM**
**Casp3**	**H9c2**	100±0.0	85±10.3	90±9.7	97±7.2
**Casp3**	**NRCM**	100±0.0	92±5.1	92±4.7	93±4.8
**Casp8**	**H9c2**	100±0.0	94±7.1	90±8.6	85±6.2
**Casp8**	**NRCM**	100±0.0	85±11.3	84±10.4	80±4.2
**AIF**	**H9c2**	100±0.0	91±10.5	84±8.1	98±7.6
**AIF**	**NRCM**	100±0.0	96±3.6	88±5.7	90±4.8
**Hyperoxia (80% O_2)**					
**dexmedetomidine**		**–**	**0.1 µM**	**1 µM**	**10 µM**
**Casp3**	**H9c2**	162±10.2	102±14.2	96±13.4	88±4.9
**Casp3**	**NRCM**	137±1.5	81±9.4	78±6.5	82±5.2
**Casp8**	**H9c2**	145±10.8	78±10.2	87±8.3	78±8.3
**Casp8**	**NRCM**	164±33.8	92±12.2	112±6.6	109±6.0
**AIF**	**H9c2**	157±14.2	90±6.8	94±7.3	85±7.3
**AIF**	**NRCM**	29±0.6	50±12.8	48±9.6	36±6.0

Data are normalized to the level of cardiomyocytes exposed to normoxia (100%) and are presented as mean (%) ± standard error of the mean (SEM). n = 6 individual experiments/group.
