# Supplementary material for: Cardioprotective Effects of Dexmedetomidine in an Oxidative-Stress In Vitro Model of Neonatal Rat Cardiomyocytes
Source: Antioxidants (Basel). 2023 Jun 2;12(6):1206. doi: 10.3390/antiox12061206 (PMC10295527; doi:10.3390/antiox12061206)
Supplement: Supplementary file 1 [file antioxidants-12-01206-s001.zip › Table S4 Quantification of autophagy.pdf]

Table S4. Quantification of autophagy factors (H9c2 and NRCM)

Hypoxia (5% O_2)					
**dexmedetomidine**		**–**	**0.1 µM**	**1 µM**	**10 µM**
**Atg5**	**H9c2**	130±5.0	91±6.2	112±8.3	96±8.3
**Atg5**	**NRCM**	51±3.8	57±3.8	66±4.8	69±4.9
**Atg12**	**H9c2**	148±12.2	91±7.1	86±14.3	94±8.7
**Atg12**	**NRCM**	46±8.0	55±5.8	66±6.2	71±8.8
**Normoxia (21% O_2)**					
**dexmedetomidine**		**–**	**0.1 µM**	**1 µM**	**10 µM**
**Atg5**	**H9c2**	100±0.0	88±8.6	89±4.1	93±3.7
**Atg5**	**NRCM**	100±0.0	80±5.8	87±7.9	86±6.9
**Atg12**	**H9c2**	100±0.0	92±5.7	87±9.1	82±9.5
**Atg12**	**NRCM**	100±0.0	85±4.6	90±5.6	85±5.8
**Hyperoxia (80% O_2)**					
**dexmedetomidine**		**–**	**0.1 µM**	**1 µM**	**10 µM**
**Atg5**	**H9c2**	137±5.9	88±5.9	86±8.7	102±11.2
**Atg5**	**NRCM**	46±6.8	58±11.4	66±12.1	52±9.7
**Atg12**	**H9c2**	142±8.6	89±6.3	81±14.3	97±11.4
**Atg12**	**NRCM**	37±9.5	62±15.9	84±6.3	69±8.2

Data are normalized to the level of cardiomyocytes exposed to normoxia (100%) and are presented as mean (%) ± standard error of the mean (SEM). n = 6 individual experiments/group.
