# Supplementary material for: Cardioprotective Effects of Dexmedetomidine in an Oxidative-Stress In Vitro Model of Neonatal Rat Cardiomyocytes
Source: Antioxidants (Basel). 2023 Jun 2;12(6):1206. doi: 10.3390/antiox12061206 (PMC10295527; doi:10.3390/antiox12061206)
Supplement: Supplementary file 1 [file antioxidants-12-01206-s001.zip › Table S5 Quantification of oxidative stress response.pdf]

Table S5. Quantification of oxidative stress response (H9c2 and NRCM)

**Hypoxia (5% O_2)**					
**dexmedetomidine**		**–**	**0.1 µM**	**1 µM**	**10 µM**
**GCLC**	**H9c2**	88±6.6	70±11.3	78±7.9	74±5.3
**GCLC**	**NRCM**	53±6.6	48±6.1	73±1.0	77±5.8
**Nrf2**	**H9c2**	96±5.3	96±1.8	95±3.5	98±3.9
**Nrf2**	**NRCM**	65±12.4	63±6.8	76±9.1	93±9.7
**Hif1α**	**H9c2**	147±3.2	85±7.7	107±5.1	108±6.9
**Hif1α**	**NRCM**	133±5.5	86±7.8	92±6.6	87±5.2
**Normoxia (21% O_2)**					
**dexmedetomidine**		**–**	**0.1 µM**	**1 µM**	**10 µM**
**GCLC**	**H9c2**	100±0.0	95±7.5	89±7.7	104±6.4
**GCLC**	**NRCM**	100±0.0	72±5.4	55±5.2	88±6.5
**Nrf2**	**H9c2**	100±0.0	76±4.5	71±5.0	75±3.3
**Nrf2**	**NRCM**	100±0.0	70±6.2	91±11.4	93±9.9
**Hif1α**	**H9c2**	100±0.0	91±5.9	74±2.5	96±7.3
**Hif1α**	**NRCM**	100±0.0	87±6.9	96±5.9	92±3.5
**Hyperoxia (80% O_2)**					
**dexmedetomidine**		**–**	**0.1 µM**	**1 µM**	**10 µM**
**GCLC**	**H9c2**	167±10.9	97±13.5	117±11.9	111±6.6
**GCLC**	**NRCM**	299±7.7	200±25.1	212±9.9	138±19.5
**Nrf2**	**H9c2**	190±2.9	70±1.7	83±5.9	96±8.6
**Nrf2**	**NRCM**	134±5.3	108±6.7	96±10.5	93±5.9
**Hif1α**	**H9c2**	103±2.7	102±5.8	80±7.9	86±5.1
**Hif1α**	**NRCM**	87±9.0	93±9.6	87±8.4	89±8.2

Data are normalized to the level of cardiomyocytes exposed to normoxia (100%) and are presented as mean (%) ± standard error of the mean (SEM). n = 6 individual experiments/group.
