# Supplementary material for: Cardioprotective Effects of Dexmedetomidine in an Oxidative-Stress In Vitro Model of Neonatal Rat Cardiomyocytes
Source: Antioxidants (Basel). 2023 Jun 2;12(6):1206. doi: 10.3390/antiox12061206 (PMC10295527; doi:10.3390/antiox12061206)
Supplement: Supplementary file 1 [file antioxidants-12-01206-s001.zip › Table S6 Quantification of tissue structure.pdf]

Table S6. Quantification of tissue structure (H9c2 and NRCM)

**Hypoxia (5% O_2)**					
**dexmedetomidine**		**–**	**0.1 µM**	**1 µM**	**10 µM**
**TNNT2**	**H9c2**	109±3.2	88±7.1	85±7.7	73±3.9
**TNNT2**	**NRCM**	82±8.5	88±6.9	95±5.8	94±8.8
**Timp1**	**H9c2**	97±4.4	98±4.3	103±6.4	91±5.9
**Timp1**	**NRCM**	83±9.0	94±6.2	97±4.3	99±5.5
**Timp2**	**H9c2**	100±5.5	86±5.1	95±4.6	97±5.2
**Timp2**	**NRCM**	68±6.0	69±5.1	79±6.7	94±6.2
**Normoxia (21% O_2)**					
**dexmedetomidine**		**–**	**0.1 µM**	**1 µM**	**10 µM**
**TNNT2**	**H9c2**	100±0.0	90±6.8	82±3.6	85±5.1
**TNNT2**	**NRCM**	100±0.0	93±8.3	110±4.6	97±8.1
**Timp1**	**H9c2**	100±0.0	92±5.2	90±7.1	96±7.2
**Timp1**	**NRCM**	100±0.0	89±5.6	107±5.7	107±6.8
**Timp2**	**H9c2**	100±0.0	93±6.2	92±7.5	105±5.7
**Timp2**	**NRCM**	100±0.0	83±4.2	93±5.7	98±2.5
**Hyperoxia (80% O_2)**					
**dexmedetomidine**		**–**	**0.1 µM**	**1 µM**	**10 µM**
**TNNT2**	**H9c2**	140±7.4	89±11.4	80±8.4	79±4.4
**TNNT2**	**NRCM**	47±6.6	58±7.5	57±5.1	39±4.6
**Timp1**	**H9c2**	140±4.2	83±8.8	93±8.0	90±8.2
**Timp1**	**NRCM**	140±6.8	90±12.5	95±8.4	80±10.3
**Timp2**	**H9c2**	143±4.1	85±6.1	115±2.7	114±2.5
**Timp2**	**NRCM**	124±3.3	87±8.4	98±7.5	89±9.7

Data are normalized to the level of cardiomyocytes exposed to normoxia (100%) and are presented as mean (%) ± standard error of the mean (SEM). n = 6 individual experiments/group.
