# Supplementary material for: Cardioprotective Effects of Dexmedetomidine in an Oxidative-Stress In Vitro Model of Neonatal Rat Cardiomyocytes
Source: Antioxidants (Basel). 2023 Jun 2;12(6):1206. doi: 10.3390/antiox12061206 (PMC10295527; doi:10.3390/antiox12061206)
Supplement: Supplementary file 1 [file antioxidants-12-01206-s001.zip › Table S7 Quantification of hippo signaling.pdf]

Table S7. Quantification of Hippo signaling (H9c2 and NRCM)

**Hypoxia (5% O_2)**					
**dexmedetomidine**		**–**	**0.1 µM**	**1 µM**	**10 µM**
**CUL7**	**H9c2**	61±5.0	74±10.4	87±8.8	101±5.6
**CUL7**	**NRCM**	47±8.5	56±8.2	89±6.9	91±8.4
**Lats2**	**H9c2**	141±7.4	79±12.9	107±2.7	108±10.3
**Lats2**	**NRCM**	122±1.1	72±6.0	84±9.4	78±8.5
**Tead1**	**H9c2**	57±7.2	58±10.7	82±5.8	90±9.1
**Tead1**	**NRCM**	68±2.9	91±10.9	96±9.3	82±6.6
**YAP1**	**H9c2**	83±2.6	80±12.8	95±5.7	93±8.5
**YAP1**	**NRCM**	84±8.4	70±6.0	103±6.1	89±3.0
**Normoxia (21% O_2)**					
**dexmedetomidine**		**–**	**0.1 µM**	**1 µM**	**10 µM**
**CUL7**	**H9c2**	100±0.0	82±11.2	91±7.8	98±9.2
**CUL7**	**NRCM**	100±0.0	77±3.2	72±9.1	73±12.2
**Lats2**	**H9c2**	100±0.0	100±9.6	94±6.5	107±7.2
**Lats2**	**NRCM**	100±0.0	84±3.6	103±4.8	112±3.6
**Tead1**	**H9c2**	100±0.0	74±4.4	78±11.6	94±6.3
**Tead1**	**NRCM**	100±0.0	82±4.4	89±5.8	94±8.9
**YAP1**	**H9c2**	100±0.0	79±12.7	84±8.9	91±8.7
**YAP1**	**NRCM**	100±0.0	80±7.7	76±2.0	97±8.1
**Hyperoxia (80% O_2)**					
**dexmedetomidine**		**–**	**0.1 µM**	**1 µM**	**10 µM**
**CUL7**	**H9c2**	48±6.1	57±8.7	91±9.8	104±3.9
**CUL7**	**NRCM**	48±10.1	70±17.2	88±11.1	86±13.7
**Lats2**	**H9c2**	148±7.6	70±9.7	82±7.6	96±9.8
**Lats2**	**NRCM**	136±5.1	84±15.1	93±15.9	103±8.2
**Tead1**	**H9c2**	59±10.6	72±5.5	93±6.5	102±4.5
**Tead1**	**NRCM**	48±12.4	91±11.7	87±5.7	79±9.7
**YAP1**	**H9c2**	159±7.1	77±10.3	87±6.6	93±8.4
**YAP1**	**NRCM**	225±32.4	118±10.9	102±16.6	101±13.0

Data are normalized to the level of cardiomyocytes exposed to normoxia (100%) and are presented as mean (%) ± standard error of the mean (SEM). n = 6 individual experiments/group.
